# DPYSL2 is a novel regulator for neural stem cell differentiation in rats: revealed by *Panax notoginseng* saponin administration

**DOI:** 10.1186/s13287-020-01652-4

**Published:** 2020-04-16

**Authors:** Liu-Lin Xiong, De-Lu Qiu, Guang-Hui Xiu, Mohammed Al-Hawwas, Ya Jiang, You-Cui Wang, Yue Hu, Li Chen, Qing-Jie Xia, Ting-Hua Wang

**Affiliations:** 1 Institute of Neurological Disease, Translational Neuroscience Center, West China Hospital, Sichuan University, Chengdu, 610041 China; 2 School of Pharmacy and Medical Sciences, Division of Health Sciences, University of South Australia, Adelaide, Australia; 3 Institute of Neuroscience, Kunming Medical University, Kunming, 650031 China

**Keywords:** Neural stem cells, DPYSL2, *Panax notoginseng* saponins, DPYSL2-knockout

## Abstract

**Background:**

The limited neuronal differentiation of the endogenous or grafted neural stem cells (NSCs) after brain injury hampers the clinic usage of NSCs. *Panax notoginseng* saponins (PNS) were extensively used for their clinical value, such as in controlling blood pressure, blood glucose, and inhibiting neuronal apoptosis and enhancing neuronal protection, but whether or not it exerts an effect in promoting neuronal differentiation of the endogenous NSCs is completely unclear and the potential underlying mechanism requires further exploration.

**Methods:**

Firstly, we determined whether PNS could successfully induce NSCs to differentiate to neurons under the serum condition. Mass spectrometry and quantitative polymerase chain reaction (Q-PCR) were then performed to screen the differentially expressed proteins (genes) between the PNS + serum and serum control group, upon which dihydropyrimidinase-like 2 (DPYSL2), a possible candidate, was then selected for the subsequent research. To further investigate the actual role of DPYSL2 in the NSC differentiation, DPYSL2-expressing lentivirus was employed to obtain DPYSL2 overexpression in NSCs. DPYSL2-knockout rats were constructed to study its effects on hippocampal neural stem cells. Immunofluorescent staining was performed to identify the differentiation direction of NSCs after 7 days from DPYSL2 transfection, as well as those from DPYSL2-knockout rats.

**Results:**

Seven differentially expressed protein spots were detected by PD Quest, and DPYSL2 was found as one of the key factors of NSC differentiation in a PNS-treated condition. The results of immunostaining further showed that mainly Tuj1 and GFAP-positive cells increased in the DPYSL2-overexpressed group, while both were depressed in the hippocampal NSCs in the DPYSL2-knockout rat.

**Conclusions:**

The present study revealed that the differentiation direction of NSCs could be enhanced through PNS administration, and the DPYSL2 is a key regulator in promoting NSC differentiation. These results not only emphasized the effect of PNS but also indicated DPYSL2 could be a novel target to enhance the NSC differentiation in future clinical trials.

## Introduction

Neural stem cells (NSCs) existing in the subventricle zone of the brain’s germinal region and the subgranular zone of the hippocampus are capable of self-renewing, proliferating, migrating, and differentiating into various types of cells within the brain and spinal cord tissue [13, 41]. NSC differentiation into neurons, astrocytes, and oligodendrocytes could replace necrotic cells resulting from injuries to promote the structural and functional repair of the brain and spinal cord [50]. However, this self-renewal is not adequate for the recovery of neurological function after brain injury [1, 32, 47]. NSCs (autologous or grafted) tend to differentiate into gliocytes rather than the neurons which are more valuable in the nervous system’s recovery [39, 51]. The development of the NSC therapy is therefore restricted, meaning the mechanisms of the NSC differentiation into neurons need to be elucidated [15]. So far, many signals and genes were demonstrably involved in the process of NSC differentiation, such as the bHLH gene, Notch signal, Wnt signal, MAPK signal, and even some micro-RNAs [5, 12, 45, 46, 54]. However, the effective regulation involving in Chinese medicine for the NSC differentiation is complex and demands exploration.

*Panax notoginseng* saponins (PNS) are one of over 200 chemical ingredients isolated from *Panax notoginseng* (Burk) F.H Chen*—*the widely used Chinese herbal medicine [17, 29]. PNS are clinically used as the Xuesaitong injection, the Xueshuantong injection, the Lulutong injection, the PNS tablet, and the Xuesaitong soft capsule [9, 40, 48]. PNS were demonstrated to have anti-inflammatory and anti-apoptotic effects contributing to the regulation of nerve cell activity and the secretion of nerve preservative agent [10, 49, 55]. In addition, recent studies have reported its preventive and therapeutic effects on the alleviation of neurological dysfunction [21, 42, 52]. In Si et al.’s study, the administration of 100 μg/ml PNS in the rat’s embryonic cortical NSC medium has demonstrated an increased number of Tuj1, GFAP, and nestin-positive cells after 4 days of culturing. Hence, conclusions were made that PNS could improve the survival status, self-renewal, proliferation, and differentiation of the rat’s embryonic cortical NSCs [37]. However, little is known about the underlying related molecular mechanism for the key factors that induce the NSCs to differentiate into neurons after PNS administration. Therefore, studying this mechanism would have tremendous value to NSC-based therapy in central nervous system diseases.

In this study, following the 100-μg/ml PNS treatment of NSCs, the proliferation and differentiation of the NSCs were observed. To explore the related molecular mechanism, mass spectrometry and Q-PCR were employed to detect the differential protein and gene expression. Then bioinformatics analysis was used to research the relatively important factors, among which DPYSL2 was found to interact with neuron markers Tubb3 (tubulin, beta 3 class III) and Numb, thereby functioning in the process of neurogenesis. Thereafter, overexpressed DPYSL2 in the NSCs revealed that DPYSL2 overexpression could induce the NSCs to differentiate into neurons, which was indicated by the increased number of Tuj1-positive cells. Meanwhile, DPYSL2-knockout rats were used to further verify DPYSL2’s function in NSCs, and the process contrarily demonstrated the diminished level of Tuj1-positive cells. This resulted in the conclusion that the exogenous application of DPYSL2 in NSCs could mediate the NSC differentiation into neurons, which could provide novel insights into NSC-based therapy in the clinical treatment of neurological disorders.

## Material and methods

### Isolation of NSCs

The NSCs were isolated from rat hippocampal tissues. Animal experimental protocols were approved by the guidelines of the Institutional Medical Experimental Animal Care Committee of Sichuan University, West China Hospital, China. After being disinfected with 75% ethanol for 2 min and anesthetized by isoflurane inhalation, the neonatal rats (1 day) were sacrificed, and the heads were immersed into D-Hanks Solution (Biohao). The hippocampal tissues of neonatal rats were harvested and were cut into several pieces (about 1 mm^3^) then suspended with 5 ml DMEM medium by pipetting. Neural-basal medium (DMEM/F-12, 1:1, Hyclone) was used to maintain the isolated cells. The medium was supplemented with 1% N_2_ (Gibco), 20 μg/L basic fibroblast growth factor (bFGF) (Invitrogen), 2 mmol/L glutamine (Invitrogen), 10,000 U/L penicillin (Hyclone), and 10 mg/L streptomycin (Hyclone). The cells were seeded onto the cell culture flasks, incubated with 5% CO_2_ at 37 °C. The culture medium was half changed every other day. The primary NSCs were maintained for 7 days for the following immunofluorescent staining and mass spectrum.

### Drug administration of the P2 NSCs

P2 NSCs were divided into four groups: normal (Nor), serum (Ser), PNS, and PNS + serum (P + S) groups. Ten percent fetal calf serum (Millipore) was added into the NSCs in the Ser group, 100 μg/ml PNS (Guangxi Wuzhou Pharmaceutical Group, Z20025652) into the PNS group, and both serum and PNS into the P + S group. Meanwhile, NSCs in the Nor group were received an equal quantity of the neural-basal medium. After 7 days, immunofluorescent staining and mass spectrometry were performed to detect the differentiation index and protein expression.

### Immunofluorescent staining

#### Cells preparation

The cells were collected by a scraper and suspended with a phosphate-buffered solution (PBS). The cell suspension was applied onto the glass slide treated by polylysine (40 g/L) (Sigma). Smears of NSCs were fixed with 4% paraformaldehyde (Sigma) for 20 min at 4 °C, followed by washing-out with PBS (0.01 mol/L) (Sigma) for three times (5 min each).

#### Immunofluorescent staining

Briefly, after washing, the cells were incubated with 0.3% TritonX-100 (Hengdailao) at 37 °C for 30 min. Then, 5% goat serum (Millipore) was added to block the nonspecific binding site and incubated at 37 °C for 30 min. Subsequently, the primary antibodies were added and incubated in the wet box at 4 °C for 20 h. The same volume of 2% goat serum (Invitrogen) was used as the negative control. The secondary antibody was added and incubated at 37 °C for 1 h. All information of the primary and secondary antibodies is shown in Table 1. Then, the sections/cells were washed 3 times with 0.01 M PBS again. Afterwards, DAPI was used to stain the nucleus. Inversed fluorescent microscope (Leica, Wetzlar, Germany) was used to observe the outcomes and capture photos. All the photos were taken in the same exposure intensity with the same setting of the Leica Microsystem. In each group (5 samples), 5 views of each sample were taken randomly. Then, cell number, area, and the length of the processes were calculated by using Image-Pro plus 6.0 software (MediaCybernetics, Silver Spring, MD, USA). The final results were shown as the average from three observers blinded to the experimental condition.

**Table 1:** The information of the antibodies

Primary antibody	Species	Dilution	Company	Secondary antibody	Species	Dilution	Company
Nestin	Rabbit	1:100	ZSGB-BIO	Alexa-594	Anti-rabbit	1:200	ZSGB-BIO
Tuj1	Mouse	1:200	Millipore	Alexa-488	Anti-mouse	1:100	ZSGB-BIO
GFAP	Rabbit	1:200	Millipore	Alexa-594	Anti-rabbit	1:200	ZSGB-BIO
DPYSL2	Rabbit	1:100	Abcam	Alexa-594	Anti-rabbit	1:200	ZSGB-BIO

### Second dimension gel electrophoresis (2D electrophoresis) and matrix-assisted laser desorption/ionization-TOF/TOF mass spectrometry (MALDI-TOF/TOFMS)

As the differentiation of NSCs only showed significance between the PNS and P + S group, so we just did 2D electrophoresis in these two groups. Cells were lysed with 1 ml lysis buffer for 10 min before 20 μg/ml DNase, 50 μg/ml RNase, and MnCl2 (metered volume with DNase). Then, after 15 min at 4 °C, samples were centrifuged at 15,000 r/min for 30 min followed by supernatants collection to new tubes. The protein concentration was determined by the Bradford Coomassie blue colorimetric assay. Thereafter, 0.02 g/ml DTT and 0.0025 μl/ml Bio-lyte were added into 1.2 mg sample solution (volume, 580 μl) and were mixed uniformly and incubated for 1 h at the room temperature prior to centrifugation at 25,000 r/min for 10 min to separate the protein samples. The supernatant was transferred into the horizontal plate and one-dimensional isoelectric focusing (IEF) was performed using IPG strips (17 cm, pH 3–10) with 50 V at low speed for half an hour, 250 V at low speed for 1 h, 500 V at rapid speed for 1.5 h, 1000 V at rapid speed for 1 h, 4000 V for 3 h, 9000 V for 3 h, 9000 V at rapid speed for 50,000 V/h, and 500 V with rapid speed for 30 min.

After equilibration for 15 min, SDS-PAGE was performed to separate the proteins further. The initial voltage was 70 V and then changed to 300 V until the bromophenol blue (BPB) crossed the band and then stopped until the BPB reached the 5 cm from the glass. Finally, the stripe was stained by Coomassie brilliant blue (CBB) G-250. Gel images were scanned for digitization (American Bio-Rad), and the differential proteins were identified by the PD Quest analysis software (Bio-Rad PD Quest Advanced 7.4). “Significant changes” was defined as “more than 1.5-fold changes in expression compared to the serum group,” and only those spots were gown through matrix-assisted laser desorption/ionization-TOF/TOF mass spectrometry (MALDI-TOF/TOFMS) analysis to identify the protein types.

### Quantitative polymerase chain reaction (Q-PCR)

Cells were collected from each group and the total RNA was extracted by using TRIzol reagent (Invitrogen) according to the manufacturer’s instruction (superfecTRI), followed by reverse transcription to complementary DNA (cDNA) (TakaRa). Q-PCR was then performed to determine the gene expression of the differential proteins which were identified by 2D electrophoresis and MALDI-TOF/TOFMS. Five genes for 6 proteins were studied in addition to β-actin that was used as an internal control; the used primers and probes were listed in Table 2. PCR was performed in a DNA thermal cycler (ABI 7300) according to the following standard protocol: 90 °C for 5 min, denaturation at 94 °C for 30 s, annealing for 30 s, and extension at 72 °C for 1 min (total 30 cycles). The relative expression levels of mRNA were calculated as standardization to β-actin by using the 2^–△△Ct^ method. Each group had 6 samples.

**Table 2:** The information of the PCR primers and probes

Genes	Primer/probe	Sequence
CRMP1/FSCN1	Forward	GGAGATTTGATAGCTCAGGA
	Reverse	GACCTTGGTGATCACAG
	Probe	CCTGGAGATGGGCATCAC
DPYSL2	Forward	GAAGGGAACTGTGGTGTAT
	Reverse	GAGGTCTCCACAGGACAG
	Probe	CCAACCACTCCAGACTTTCTC
GRP78	Forward	GAGTTCTTCAATGGCAAGGAG
	Reverse	CCTCCCACAGTTTCAATACCA
	Probe	CTGTCCAGGCTGGTGTCCTC
HSP90/TRA	Forward	CAAATGCTTCTGATGCTTTAG
	Reverse	CTCCTCTCTGGTCATTCCTACA
	Probe	CCTGCTGCATGTCACAGACAC
LDHB	Forward	CCTCAGATCGTCAAGTACAG
	Reverse	GCAGCTGCTGGGATGAAT
	Probe	CCACTGGGTTGGAAACCAC
β-actin	Forward	GAAGATCAAGATCATTGCTCCT
	Reverse	TACTCCTGCTTGCTGATCCA
	Probe	CTGTCCACCTTCCAGCAGA

### Lentivirus transduction

#### ORF-vector verification by enzyme digest and electrophoresis and recombinant lentivirus production

Gene sequence of DPYSL2/CRMP2 was acquired from NCBI, and then PCR and electrophoresis were performed to amplify and purify the DPYSL2 gene from rats’ brain. The target DNA fragment was sent to GeneCopoeia Company to construct overexpression recombinant vector (ORF-vector).

The lentiviral particles were generated by the standardized protocol using highly purified plasmids and EndoFectin-Lenti™ and TiterBoost™ reagents. Then, lentiviral stocks were stored at − 80 °C to maintain the activity.

### NSC transfection with lentivirus

In this experiment, the cultured NSCs were divided into the following three groups: Nor, eGFP, and DPYSL2 groups. In brief, in the DPYSL2 group, the P2 NSCs were transfected with the lentivirus and were transfected to up to 20 MOI with 10% polybrene in the 96-well plate; the eGFP group was treated with the same titer of the simple eGFP-lentivirus and the Nor group was treated with equal quantity of the Neural-basal medium. After 12 h incubation at 37 °C, the neural-basal medium was changed with a fresh one, and then 3- and 7-day photos were taken with the Inversed Fluorescent Microscope (Leica, Wetzlar, Germany). In addition, the positive cell number in each group was calculated by using Image-Pro plus 6.0 software (MediaCybernetics, Silver Spring, MD, USA). Five views of five samples were randomly considered for each group. At last, the average from three observers blind to the experimental condition was determined as the final results.

### Construction of a DPYSL2-knockout rat

CRISPR/CAS-mediated genome engineering was applied to construct DPYSL2-KO rats which were produced and provided by Cyagen (Guangzhou, China). Two targets were designed for DPYSL2, and single gRNA was prepared by synthesizing two pairs of oligonucleotide chains (CAACGAGTCCTTACGGACAA and CCCTGTACCTGTTAAACGTG). Then, more KO rats were reproduced by mating and were detected by genotyping.

### Genotype identification

Tail tips were collected and numbered for neonatal rats at 7–10 days. Then, Transgen’s genomic DNA extraction kit (ee101-12) was used to extract rats’ genomic DNA and were further detected by PCR with the amplification primer:

Rat DPYSL2 forward: 5′-ACTGAGCAGGTTCAGTCCGTGC-3′.

Rat DPYSL2 reverse: 5′-ACTTGTGGTGGAAGCTCTGACTCCC-3′.

The PCR amplification reaction system was conducted with 10 μl PCR master mix: 0.6 μl upstream primers, 0.6 μl downstream, 3 μl DNA template, and 5.8 μl water. The thermal cycling conditions were performed as initial denaturation at 94 °C for 5 min; 35 cycles of denaturation at 94 °C for 30 s; annealing at 60 °C for 30s, with 1 min elongation at 72 °C; and followed by final elongation at 72 °C for 10 min. Subsequently, the agarose gel electrophoresis system was applied to visualize the final genotype detection under U.V after 55 min electrophoresis at 150 V.

### Primary hippocampal stem cells cultures

The hippocampal tissues of neonatal rats were harvested following the successful construction of DPYSL2-KO rats, and then they were processed into cellular suspension. Afterwards, neural-basal medium (DMEM/F-12, 1:1, Hyclone) was supplemented with 1% N_2_ (Gibco), 20 μg/l basic fibroblast growth factor (bFGF) (Invitrogen), 2 mmol/l glutamine (Invitrogen), 10,000 U/l penicillin (Hyclone), and 10 mg/l streptomycin (Hyclone) and then seeded onto the cell culture flasks and incubated with 5% CO_2_ at 37 °C every other day. The primary hippocampal stem cells were kept for immunofluorescent staining on the 7th culturing day.

### Statistical analysis

All the data were analyzed by using one-way analysis of variance (ANOVA) with SPSS 19.0 and presented as the means ± SD. *P* < 0.05 was considered statistically significant, *P < 0.05, **P < 0.01, and ***P < 0.001.

## Results

### Observation and identification of the cultured NSCs

**Fig. 1:**
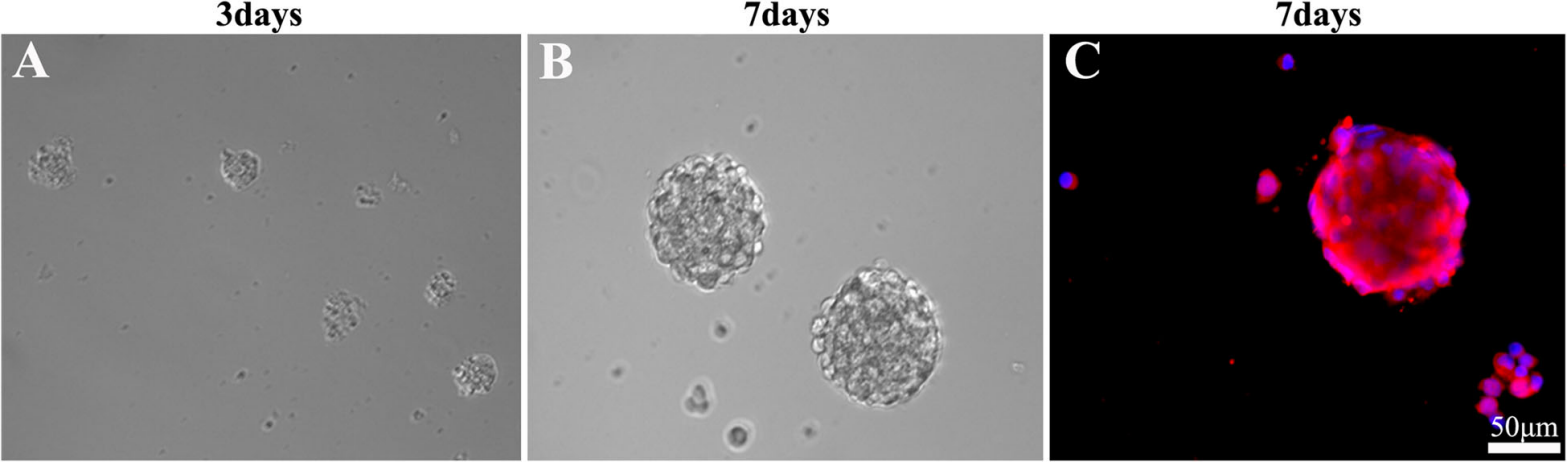
Culture and identification of NSCs. **a**, **b** The NSCs culture on days 3 and 7. **c** The immunofluorescent staining of nestin in the cultured NSCs on day 7. DAPI stained the cell nucleus (blue) and nestin stained the NSCs, which emitted positive red immunofluorescence. Scale bar, 50 μm. NSCs, neural stem cells

The cells isolated from the neonatal rats (1 p) turned out to be spheres consisting of several tens of cellular clumps on 2–3 days after isolation and showed the fluctuant growth state (Fig. 1a). Five days following culture, they gradually turned into neurospheres, each containing tens and hundreds of cells. Then the subculture increases the number of secondary neurospheres which were harvested after 7 days. The morphology of secondary neurospheres was similar to that of the primary ones (Fig. 1b), which were identified as nestin-positive cells (red) by immunofluorescence staining (Fig. 1c).

### PNS could induce NSCs to differentiate into neurons in the serum condition

**Fig. 2:**
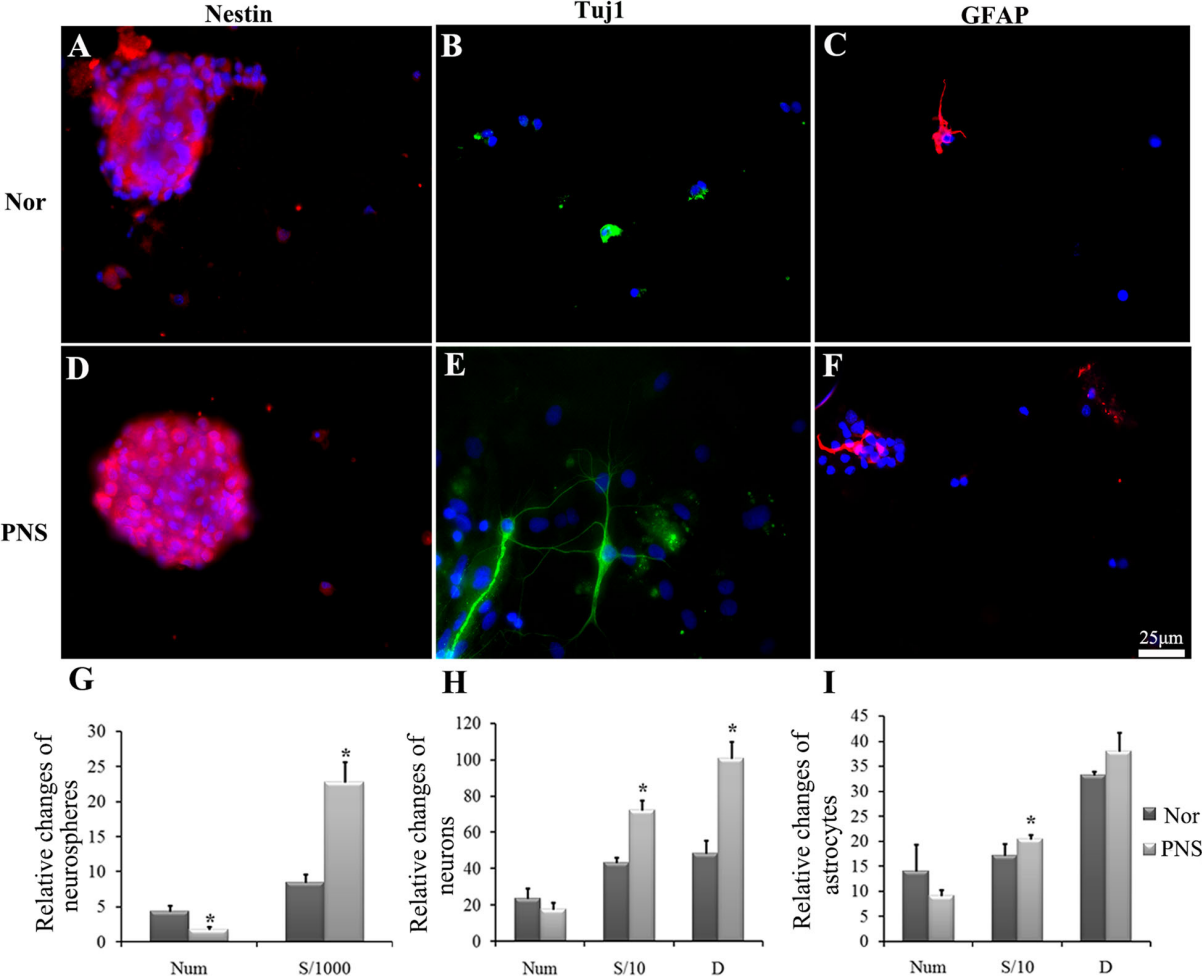
The proliferation and differentiation of the cultured NSCs in the normal and PNS groups. **a**–**d**, **b**–**e**, **c**–**f** The immunofluorescent staining of nestin (red immunofluorescence), Tuj1 (green immunofluorescence), and GFAP (red immunofluorescence) in the normal and PNS group, respectively. DAPI was used to stain the cell nucleus (blue). Scale bar, 25 μm. **g**–**i** The representative bar graphs of proliferation and differentiation of NSCs or its differentiated states in the normal and PNS groups. Data were presented as means ± SD. Each group contained 5 samples. *P < 0.05 versus Nor group. Nor, normal group; PNS, *Panax notoginseng* saponins group; Num, the number of the cells/neuronspheres; S, cell area. S/1000, S/10represents the area value divided 1000 and 10, respectively; D, the length of the synapse

The P2 NSCs were cultured in the neural basal medium supplemented with PNS for 7 days. Then, nestin, Tuj1, and GFAP were used to label the NSCs, neuron, and astrocytes, respectively. The PNS has shown to be an excellent nutrition supplement to either the neurospheres or differentiated cells, indicated by the increased area of the NSCs, neurons, and astrocytes and the length of the processes of neurons and astrocytes compared to the normal group (*P <* 0.05) (Fig. 2b, e, h, c, f, i). Also, the number of neurospheres decreased significantly (*P* < 0.05) compared to the normal group. However, there were no significant changes in the NSC differentiation (the number of neurons or astrocytes) between the normal and PNS group (Fig. 2h, i).

**Fig. 3:**
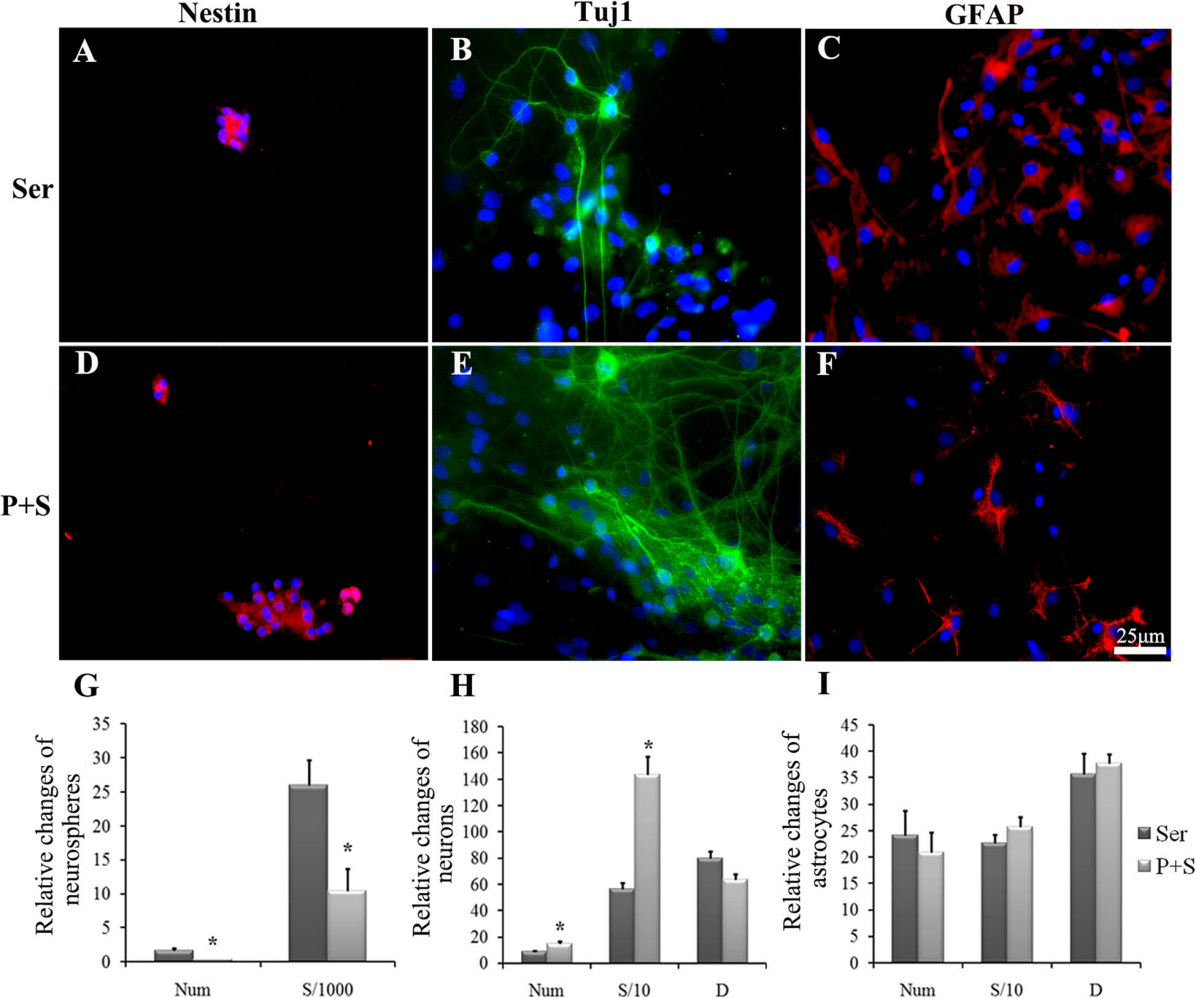
The proliferation and differentiation of the cultured NSCs. **a**–**d**, **b**–**e**, **c**–**f** The immunofluorescent staining of nestin (red immunofluorescence), Tuj1 (green immunofluorescence), and GFAP (red immunofluorescence) in the serum and PNS + serum group. DAPI was used to stain the cell nucleus (blue). Scale bar, 25 μm. **g**–**i** The representative bar graphs of the proliferation and differentiation of NSCs or its differentiated states in the serum and PNS + serum groups. Data were presented as means ± SD. Each group contained 5 samples. *P < 0.05 versus Ser group. Ser, Serum group; P + S, PNS + serum group; Num, the number of the cells/neuronspheres; S, cell area; D, the length of the synapse. S/1000, S/10 represent the area value divided 1000 and 10, respectively

After adding 10% FBS into the medium, different effects of PNS were shown on the NSC proliferation and differentiation. Although there were few neurons in the group of serum alone, the number of Tuj1-positive cells (green) increased markedly in the P + S group (9 ± 1.53 versus 14.56 ± 4.30, *P* < 0.05) (Fig. 3b, e, h). In addition, the number of neurospheres decreased significantly in the P + S group, as compared to that of the Ser group (1.63 ± 0.92 versus 0.38 ± 0.12, *P* < 0.05) (Fig. 3a, d, g), whereas there were no significant changes in the number of GFAP-positive cells in both groups (Fig. 3c, f, i). These indicate that PNS could induce NSCs to differentiate neurons without increasing the differentiation rate of astrocytes under the serum condition. Also, the enlarged cell area and reduced length of the process were considered as evidence for the effect of PNS as a supplement to the differentiated neurons (Fig. 3h, i).

### Identification of different protein expression in the Ser and P + S group by 2D electrophoresis and MALDI-TOF/MS

The differentially expressed proteins in the NSCs of the two groups were screened by the proteomic technology which was performed 3 times repeatedly to affirm the accuracy of the result. Finally, 230 (Ser group) and 287 (P + S group) protein spots were detected by PD Quest, respectively.

**Fig. 4:**
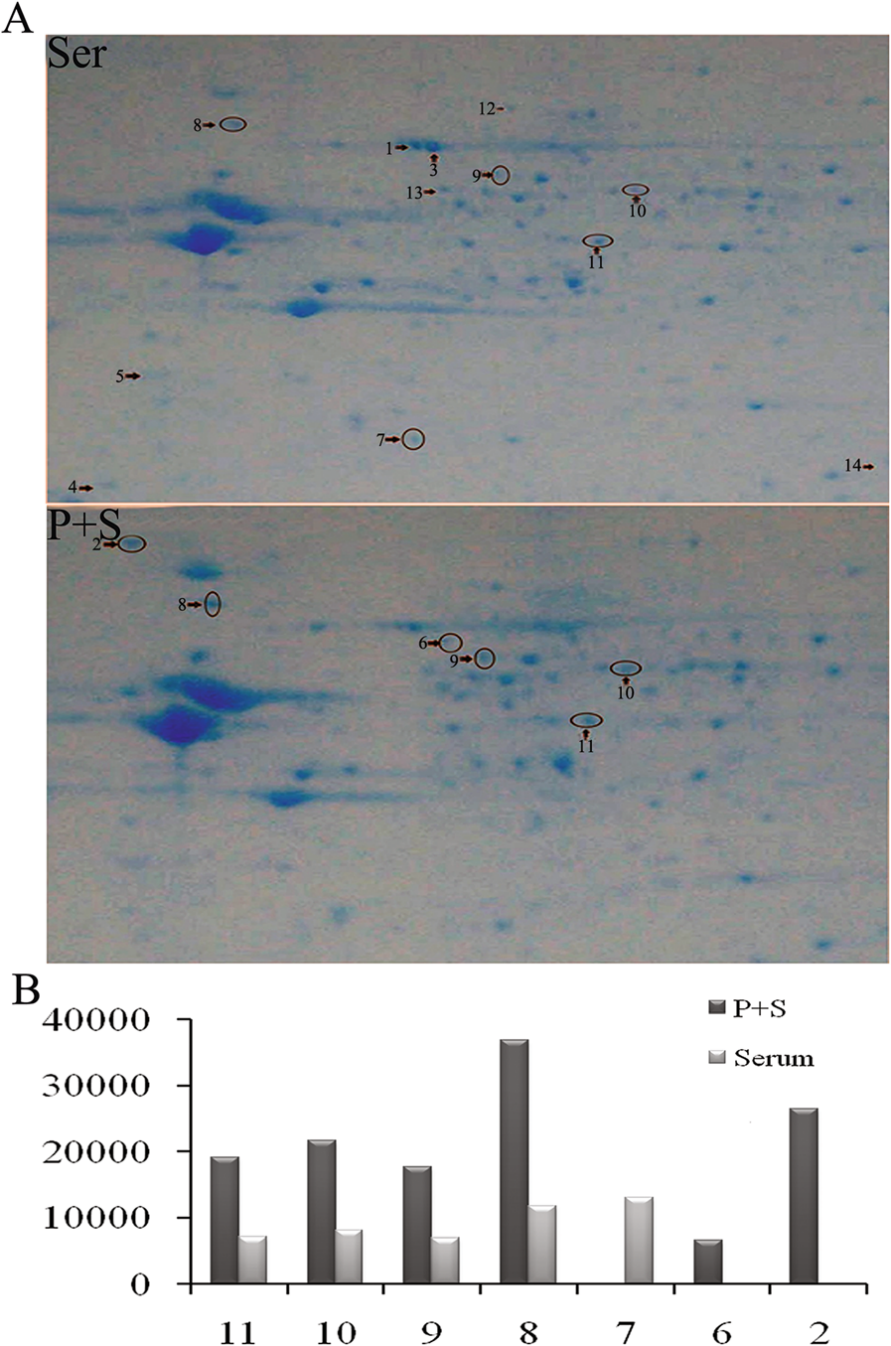
The protein expression detected by two-dimensional (2-DE) gel electrophoresis. **a** The 2-DE gel images of the cultured NSCs on day 7. The black arrows with numbers were the differently expressed proteins with significance. **b** The protein levels between the P + S and Ser group. Subcellular location and biological function were based on the database of ExPASy and KEGG pathway. Score, Ions score

The isoelectric points of the detected protein spots were mostly distributed in the pH of 4–9 and were located in the range of 10–90 kDa. The match rate of the serum group to the P + S group reached to 42%. Among these protein spots, seven differentially expressed protein spots were detected by PD Quest (Fig. 4a). Two spots were new (spot no. 2 and 6), one was missed (spot no. 7), and four were unregulated (spot no. 8, 9, 10, and 11) in the P + S group. The differences of the expression levels between the two groups were beyond 1.5 times (Fig. 4b). Then the protein types of these seven were identified successfully by MALDI-TOF/MS and PMF as follows: tumor rejection antigen/heat shock protein 90 kDa β, member 1 (TRA/HSP90-B, Spot NO.2), dihydropyrimidinase-like 2 (DPYSL2/CRMP-2, spot no. 6 and 9), lactate dehydrogenase 2 B chain (LDH-B, spot no. 7), 78 kDa glucose-regulated protein precursor (GRP 78, spot no. 8), collapsin response mediator protein 1(CRMP-1, spot no. 10), and Fscn1 protein (Fscn1, spot no. 11).

### DPYSL2 was identified as the key protein in the P + S group

**Fig. 5:**
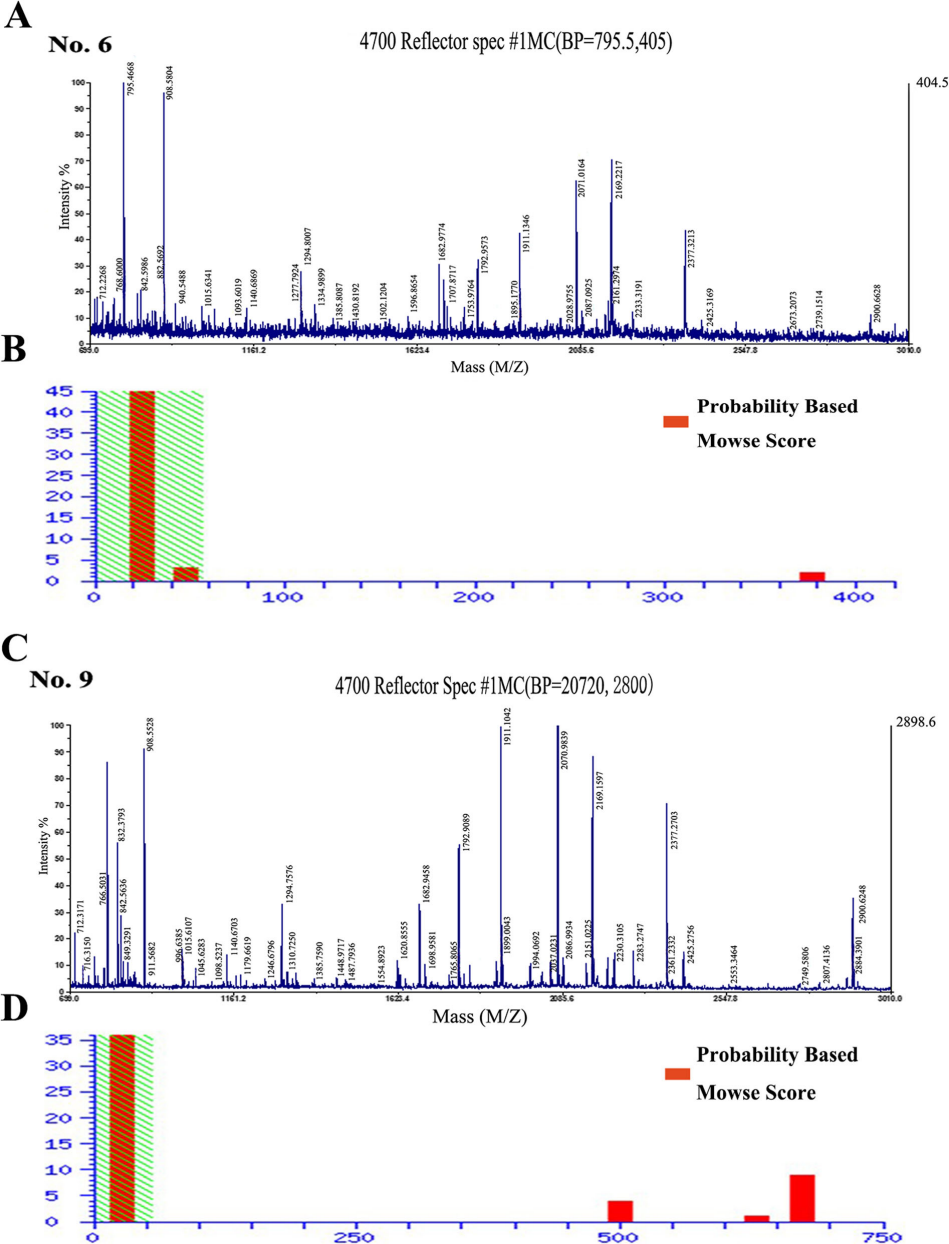
The information of the DPYSL2/CRMP2 in the two-dimensional (2-DE) gelelectrophoresis. **a**–**c** The PMF of the DPYSL2. **b**–**d** The Ions score of protein spot of DPYSL2 by MASCOT software

Two of these seven protein spots were identified as DPYSL2 (no. 6 and 9). One was new and the other was unregulated in the P + S group compared to the Ser group. The PMF of these 2 spots was shown (Fig. 5a, c), and the probability-based Mowse scores were 377 and 681 (− 10 × log (*P*); *P*, the probability of the observed match is a random event), respectively (Fig. 5b, d). Spot no. 9 exhibited higher scores relative to spot no. 6 (Fig. 5c, d). The coverage of the amino acid sequence in the DPYSL2 was 25% for spot no. 6 and 47% for spot no. 9.

### The changes of mRNA expression levels of DPYSL2 and CRMP1 in different experimental groups

**Fig. 6:**
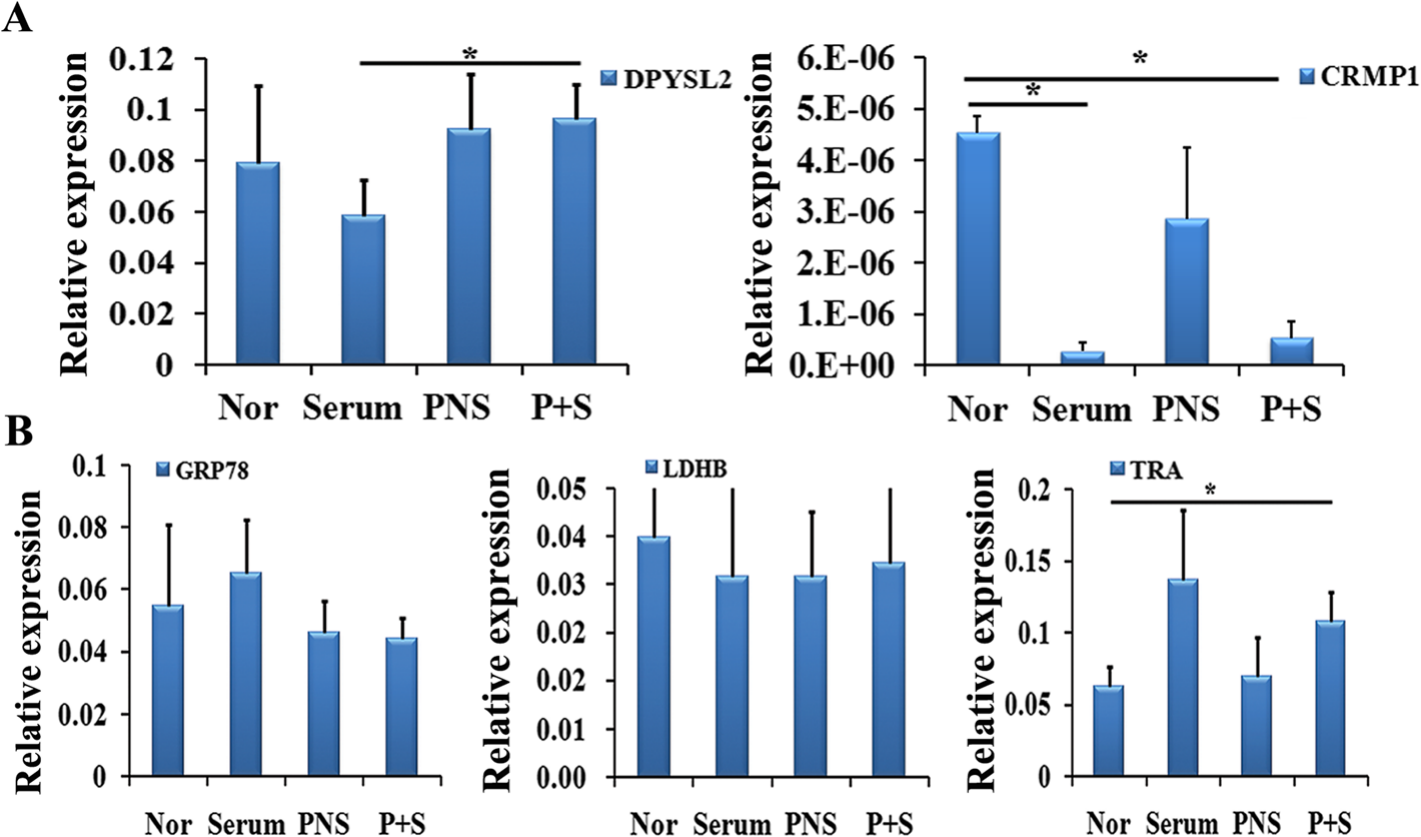
The gene expression level (standardized CT) of the identified proteins in the four groups by Q-PCR. **a** The results of DPYSL2 and CRMP1 by Q-PCR in the four groups. **b** The results of other detected genes by Q-PCR in the four groups. Data were presented as means ± SD. *P < 0.05. Each group contained 6 samples

In order to validate the result of proteomics, Q-PCR was performed to detect the genetic expression of these 6 proteins (DPYSL2 has 2 protein spots) in the four groups (normal, serum alone, PNS alone, and PNS + serum). The expression of DPYSL2 and CRMP1 aligned with the protein data (Fig. 6a). Delightedly, the calibrated CT value of DPYSL2 in the normal group was decreased to 0.08, in the serum group, and significantly increasing in the PNS and P + S groups. In addition, there was a significant increase between the serum and P + S group (0.056 versus 0.096, *P* < 0.05). Although the CRMP1 gene expression changed consistently with its protein level in the P + S and serum group mostly, its expression in each group was low. The expression levels of the other genes under the study are shown in Fig. 6b. To sum up, we speculated the possible important role of DPYSL2 in the NSC differentiation.

### The possible role of DPYSL2 from bioinformatics prediction

**Fig. 7:**
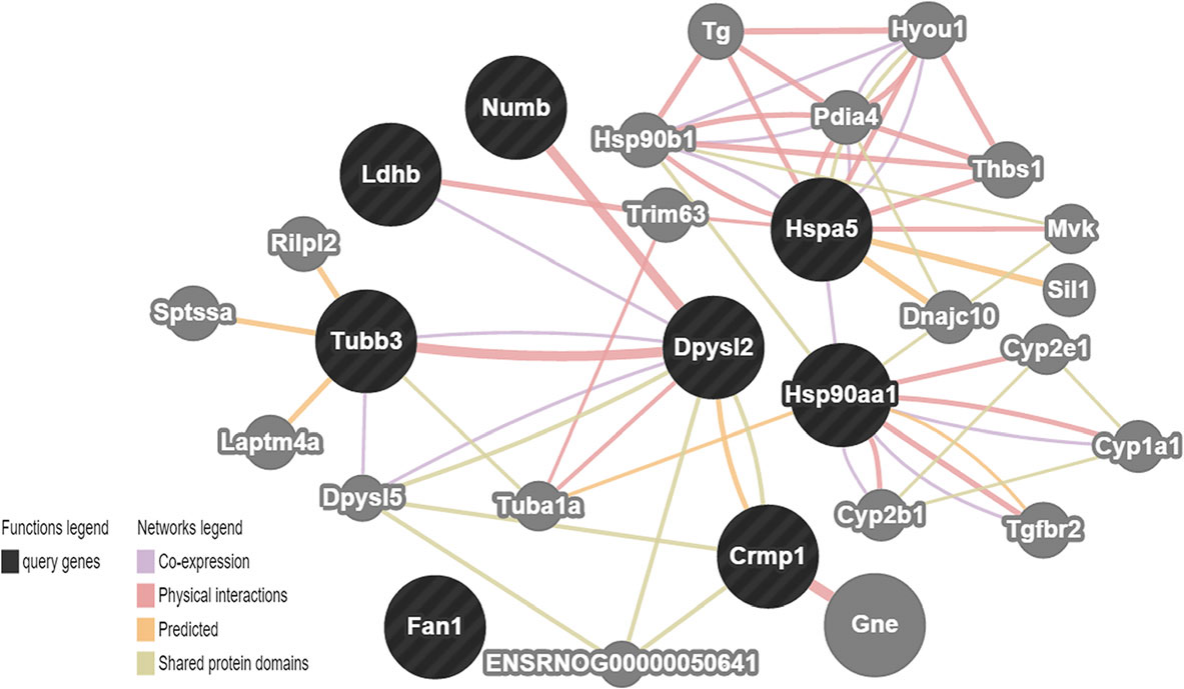
Correlation analysis of the factors detected by GeneMANIA**.** Through searching for http://www.genemania.org/, we found that DPYSL2 was encoded to a family of collapsin response mediator protein (CRMP). Moreover, it has some physical interaction with Tubb3 (tubulin, beta 3 class III) and Numb

The known interrelation of the deferential proteins was built by using GeneMANIA (http://www.genemania.org/) (Fig. 7). Delightedly, we have found that DPYSL2 was encoded to a family of collapsin response mediator protein (CRMP) which was associated with microtubule assembly and synaptic growth [25]. Moreover, DPYSL2 has revealed physical interaction with Tubb3 (tubulin, beta 3 class III) and Numb. The former is a neuron marker while the latter plays a role in the process of neurogenesis [35, 44]. Furthermore, numerous studies found that DPYSL2 plays an important role in the neuronal differentiation and polarity to further enhance axon outgrowth and guidance [18, 23, 24].

Altogether, DPYSL2 expression was enhanced in the P + S group compared to that in the serum group at both gene and protein levels. To sum up, DPYSL2 was selected as our target gene for NSC differentiation into neuron.

### Identification of the DPYSL2-ORF recombinant

**Fig. 8:**
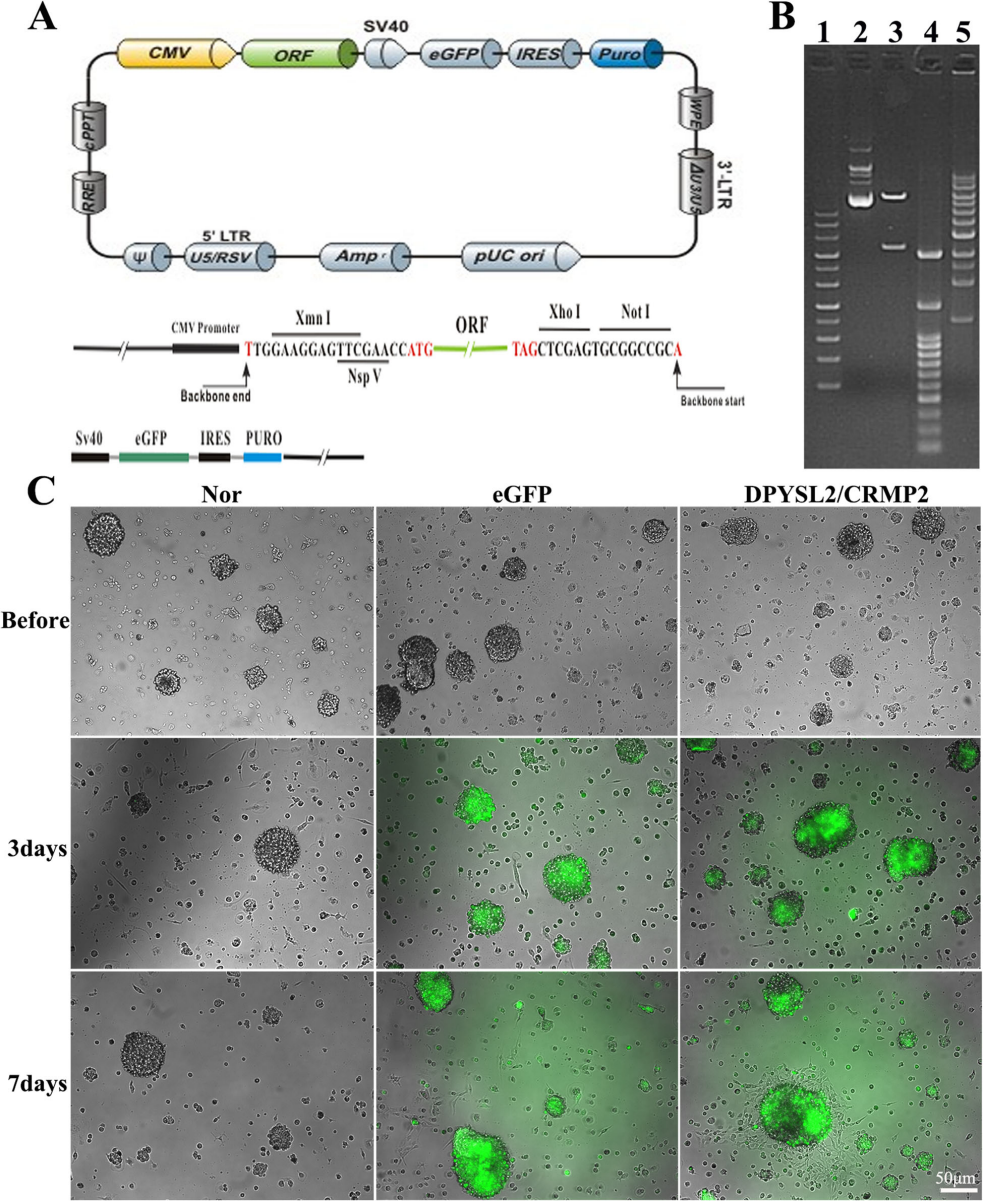
Construction and identification of the ORF-DPYSL2 lentivirus. **a** Information of the EX-Rn10192-Lv201recombinant. **b** Enzyme digestion products of EX-Rn10192-Lv201recombinant, separated by 3% agarosegel electrophoresis. Lane 1, 4, and 5: DNA Ladder. Lane 2: EX-Rn10192-Lv201 plasmid. Lane 3: EX-Rn10192-Lv201 plasmid digested by NspVandXhoI. **c** Represent NSCs pictures taken by inversed fluorescent microscope before and at 3 and 7 days post transfection. Green: eGFP positive cells. Scale bar = 50 μm. Nor, normal group, neural basal medium addition; eGFP, eGFP group, eGFP-lentivirus transfection; DPYSL2, DPYSL2 group, DPYSL2-ORF vector transfection

DPYSL2-ORF plasmid was designed and purchased from GeneCopoeia (EX-Rn10192-Lv121, 10,372 bp, *Rattus norvegicus*). The information of the plasmid vector and part of the restriction enzymes which could cleave EX-Rn10192-Lv121 is shown in Fig. 8a. Then the vector was digested by Xmn I and Not I restriction enzyme and run on 1.0% agarose gel. It was confirmed that clone EX-Rn10192-Lv201 was carried out correctly from the enzyme digestion results (Fig. 8b). Hence, lentivirus was transfected to the rats’ NSCs to the titer of 20 MOI in accordance with instruction manual, and photos were taken at 3 and 7 days post transfection. Enhanced green fluorescent protein (eGFP) could be visualized as green glow under the fluorescent microscope, representing the lentivirus were successfully replicated in the NSCs (Fig. 8c). Also, we could observe that the cells gradually grow to the sphere with prolonged time, especially in the DPYSL2 group (Fig. 8c).

### DPYSL2 overexpression in the NSCs could promote the differentiation toward neurons more than the astrocytes

**Fig. 9:**
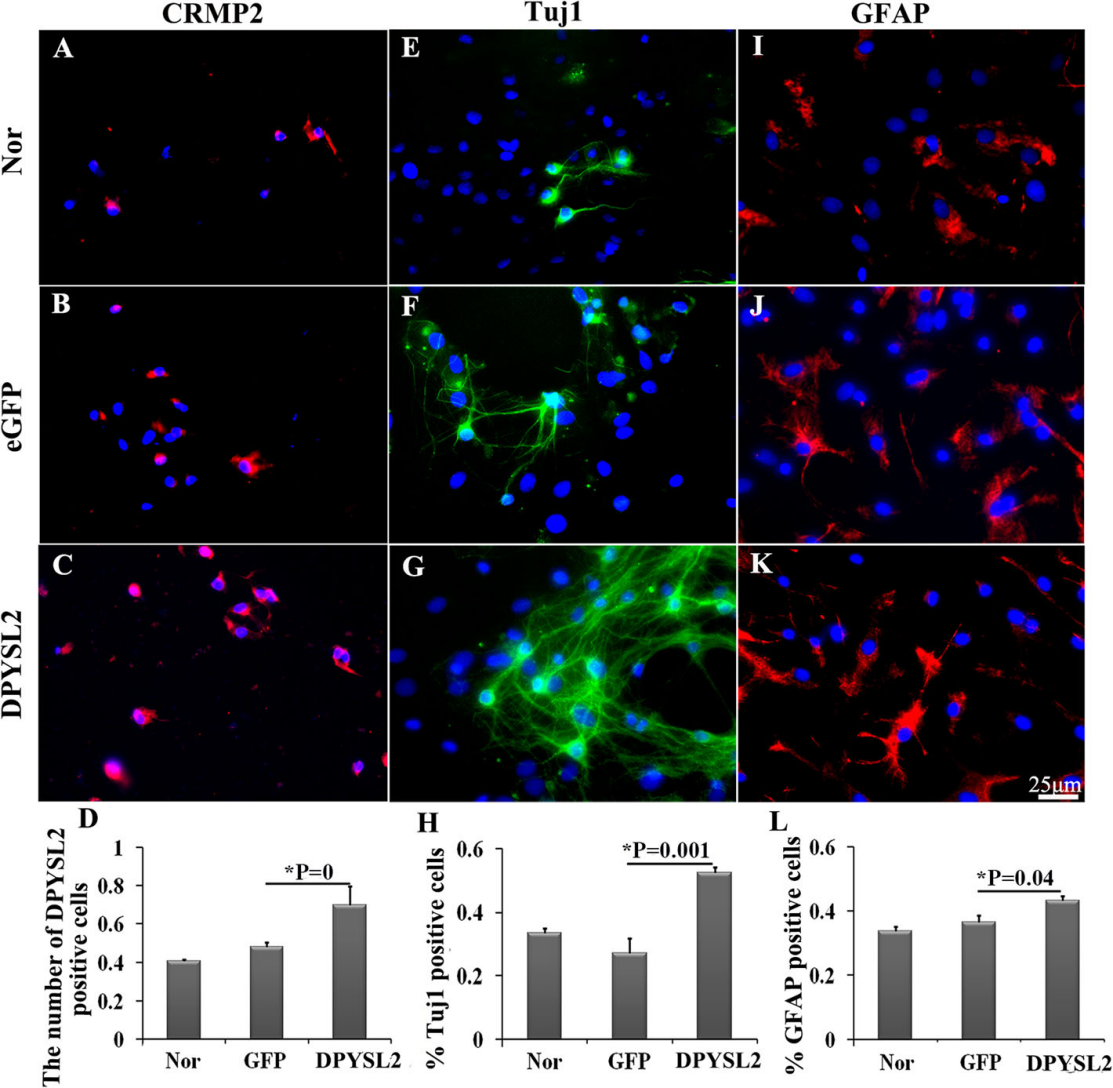
The detection of NSCs differentiation toward neurons and astrocytes after DPYSL2-ORF transfection. **a**–**c**, **e**–**g**, **i**–**k** The immunofluorescent staining of DPYSL2 (red immunofluorescence), Tuj1 (green immunofluorescence), and GFAP (red immunofluorescence), respectively, in the three groups (Nor, eGFP, DPYSL2) at 7 days after transfection, DAPI stained the cell nucleus (blue). **d**, **h**, **l** The representative bar graphs for the ratio of the positive cells in the three groups. Data were presented as means ± SD. *P < 0.05. Scale bar, 25 μm. Each group contained 5 samples

After 7 days, the transfected NSCs were collected to investigate the differentiation by immunofluorescent staining. Before identification, the immunofluorescence of DPYSL2 was used to confirm the effective transfection of DPYSL2-ORF into the NSCs. As the lentivirus was a dual promoter vector, the expression of the eGFP indicates that the lentivirus has been transfected into the NSCs instead of reflecting the gene overexpression. The positive-immunofluorescent expression of DPYSL2 was shown (Fig. 9a–c), which was enhanced in the DPYSL2 overexpressed group compared to the eGFP group (0.700 ± 0.10 versus 0.483 ± 0.02, *P* < 0.05) (Fig. 9d), demonstrating that the DPYSL2 gene was successfully overexpressed.

Interestingly, the number of the Tuj1-positive cells has increased significantly in the DPYSL2 group compared to that of the eGFP group (0.525 ± 0.02 versus 0.271 ± 0.05, *P* < 0.05). No significant change between the eGFP group and the normal group was exhibited (*P* > 0.05) (Fig. 9e–h). Similar tendencies have appeared in the immunofluorescence of the GFAP (0.431 ± 0.02 versus 0.365 ± 0.02, *P* < 0.05) (Fig. 9i–l); however, it was less obvious as compared to the Tuj1. Altogether, overexpressing DPYSL2 in rats’ NSCs could promote the NSC differentiation, especially to the neurons.

### DPYSL2 knockout suppressed the differentiation of primary hippocampal stem cells

**Fig. 10:**
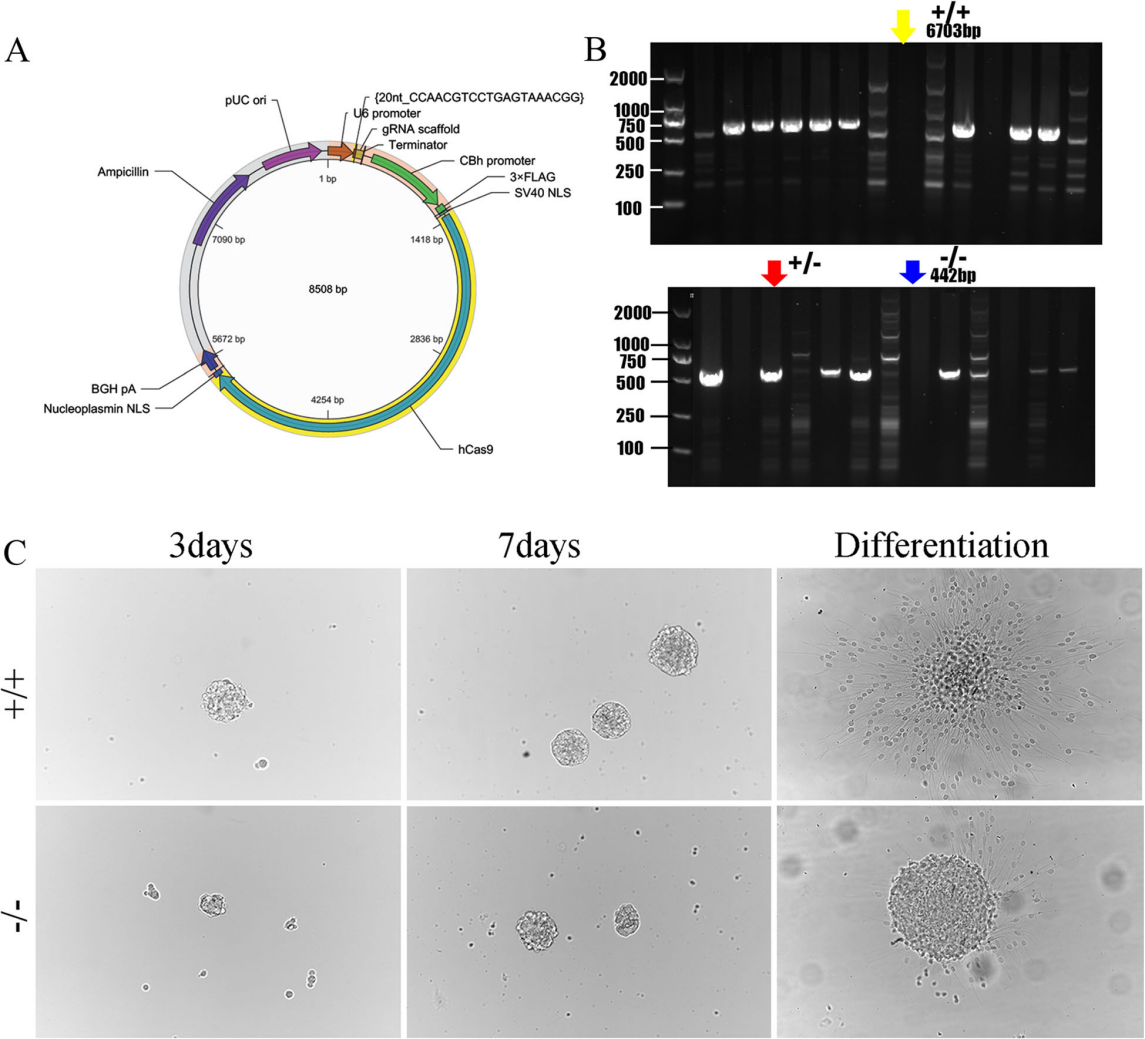
The impact of DPYSL2 knockout on the NSC differentiation into neurons. **a**–**b** DPYSL2-knockout rats plasmid construction and DPYSL2 expression identification. **c** Images of cultured hippocampal NSCs from WT (+/+) and KO (−/−) groups at 3 and 7 day as well as differentiation status. +/+, wild type rats; −/−, DPYSL2-knockout rats

To further verify the function of DPSL2 in NSCs, we constructed DPYSL2-knockout rats (Fig. 10a, b) and collected their primary hippocampal NSCs. Bright filed images of primary hippocampal NSCs have demonstrated regression in the differentiation after knocking out DPYSL2 expression indicated by the diminished number of neurospheres in the DPYSL2-knockout group compared to that in the WT group (Fig. 10c).

**Fig. 11:**
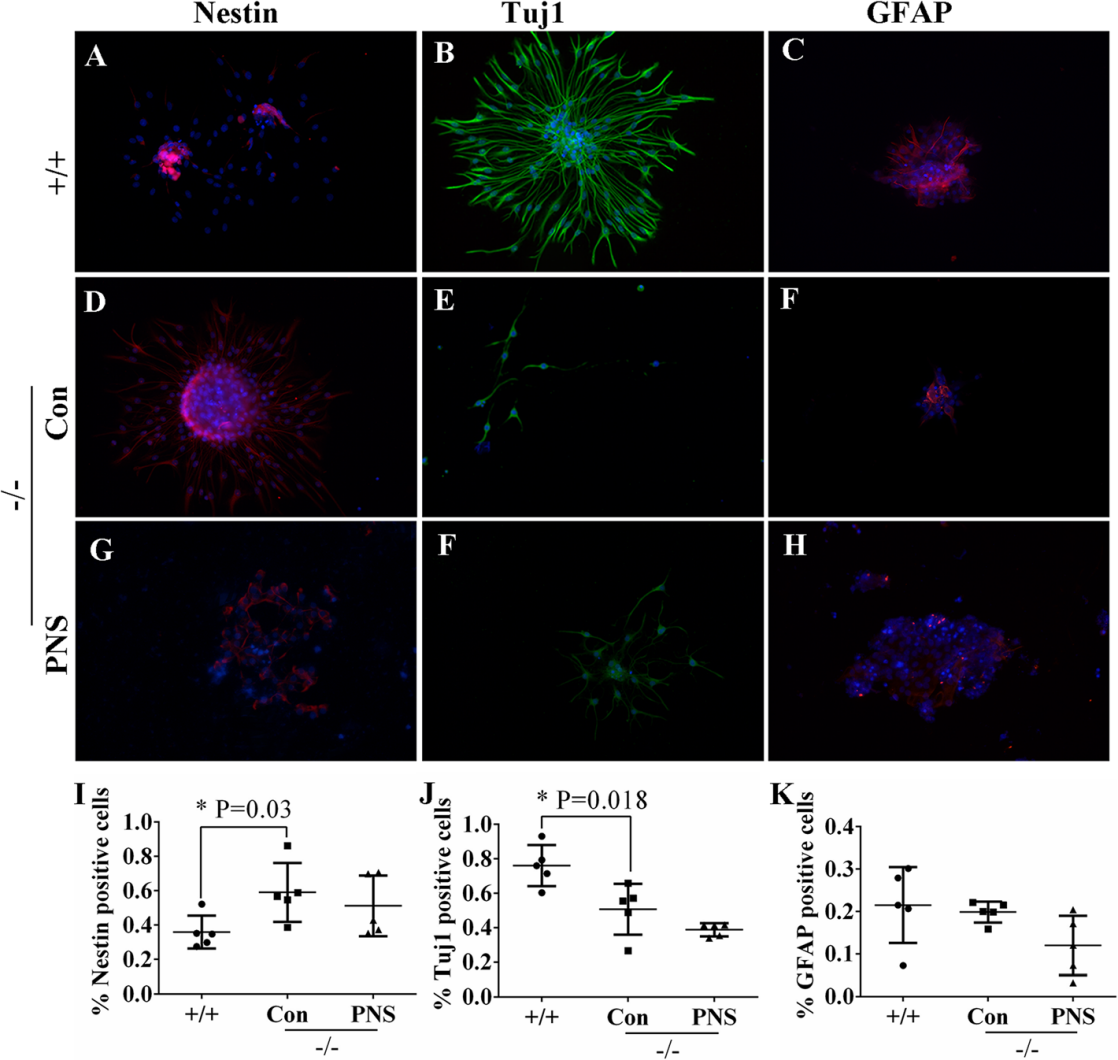
The impact of DPYSL2 knockout on the NSCs differentiation into neurons by immunofluorescent staining. The immunofluorescent staining of **a**, **d**, **g** nestin (red immunofluorescence), **b**, **e**, **f** Tuj1 (green immunofluorescence), and **c**, **f**, **h** GFAP (red immunofluorescence) in the +/+, DPYSL2^−/−^ Con group and DPYSL2^−/−^ PNS groups. DAPI was used to stain the cell nucleus (blue). Scale bar, 25 μm. **i**–**k** The representative bar graphs of the positive cells of nestin, Tuj1, and GFAP in these three groups. Data were presented as means ± SD. Each group contained 5 samples. *P < 0.05 versus DPYSL2^−/−^ Con group. Con, control group; PNS, *Panax notoginseng* saponins; +/+, wild type rats; −/−, DPYSL2-knockout rats

As shown, there were few nestin-positive cells in the WT group (Fig. 11a). The number of which (pink) increased markedly in the DPYSL2^−/−^Con group (*P* = 0.03) while decreased slightly in the DPYSL2^−/−^PNS group (Fig. 11d, g, i). In addition, the number of Tuj1-positive cells decreased significantly in the DPYSL2^−/−^Con group, as compared to the WT group (*P* = 0.018); nevertheless, no significant difference was found between the DPYSL2^−/−^Con and DPYSL2^−/−^PNS groups (Fig. 11b, e, f, j). Moreover, there was no significant variation in the number of GFAP-positive cells among these three groups (Fig. 11c, i, h, k). Thus, we have concluded that DPYSL2 knockout could hamper the differentiation of NSCs into neurons and did not impact the differentiation rate of astrocytes, although PNS seldom showed significant influence on the amount of these three kinds of cells but exhibited nutritious efficacy to the differentiated neurons, revealed by cell area and the reduced length of the process following of PNS administration (Fig. 11g, f, h).

## Discussion

Three main points have emerged from this study. First, PNS induces NSC differentiation into neurons under the serum condition in vitro. The underlying molecular mechanism for DPYSL2 upregulation was indicated by proteomic analysis. Second, overexpressing DPYSL2 in the rats’ NSCs induces differentiation into the neurons more than the astrocytes. Third, knocking out DPYSL2 hindered the differentiation capability of NSCs into neurons. Altogether, these results may provide highly valuable for NSC-based treatment in nervous system diseases.

### DPYSL2 was selected as a main mediator for the NSC differentiation into neurons under PNS condition

Our results from this study show that PNS enhances NSC differentiation into neurons in the serum condition and promotes the growth of the differentiated neurons. For some nervous system diseases or brain injuries, the neurons’ restoration may play the key role in both functional recovery and the prognosis [39, 51]. As a result of the limited capacity of self-compensation in the body, exogenous NSC transplantation was used to treat some nervous system diseases. This approach had positive effects in some cases, as previously reported [14, 31]. To date, however, the limited differentiation of NSCs into neurons has restricted the treatment by NSC transplantation, meaning a mechanism to induce NSC conversion to neurons is in demand. PNS has been used extensively in the neurology departments of Chinese hospitals to improve blood circulation. Some previous research showed that PNS administration could upregulate the expression of neurotrophic factors [11], thereby enhancing cerebral neuronal protection after ischemia [33]. Moreover, in the Alzheimer’s disease model, PNS exhibited an ability to reduce reactive oxygen species and suppress stress-activated MAPK signaling pathways, ultimately inhibiting neuronal apoptosis [30]. Mounting evidence revealed the effect of PNS to increase Tuj1 and GFAP expression in a rat’s hippocampal NSC [37, 38]. However, the related molecular mechanism for PNS’ effect on neurons still requires further clarification.

With the goal of exploring the possible molecular mechanisms, 2D electrophoresis and Q-PCR were employed in the PNS and PNS + serum group. DPYSL2 stood out from the identified differentially expressed proteins in these two groups. Moreover, DPYSL2/CRMP2, a microtubule-binding phosphoprotein involved in schizophrenia pathogenesis, was identified as a key factor involved in the NSC differentiation into neurons, promoted by PNS administration. CRMP2 is a crucial regulator of cytoskeletal dynamics facilitating axonal outgrowth and neuronal connectivity [18]. Negative alterations of DPYSL2/CRMP-2 were found to exert prominent effects on neuronal homeostasis and phenotype and may be partially responsible for the observed neurodegeneration upon chaperone-mediated autophagy impairment in vivo [6]. DPYSL2 was previously reported as being a neurite-guiding factor, with overexpressing DPYSL2 promoting dendritic growth [4]. The hyperphosphorylation of DPYSL2 may induce neuronal apoptosis in the development of Alzheimer’s disease [2, 8]. Additionally, Akama revealed in 2011 that DPYSLY2 was upregulated in the process of NSCs differentiating into neurons by proteomic identification in the cynomolgus monkey [2]. Later, it was emphasized that preconditioning with *Ginkgo biloba* (EGb 761(R)) promoted neuroprotection through HO1 and CRMP2 in rats with brain ischemia [34]. Moreover, DPYSL2 was present as an early neural differentiation regulator mediated by bone morphogenetic protein 4 (BMP-4), of which the activation could suppress the expression of DPYSL2 [16]. In addition, the high level of BMP-4 could induce the NSCs to remain in a quiescent state [43]. However, some investigations highlighted the exact role of DPYSL2 in the differentiation of NSCs, which is discussed in the current study.

### DPYSL2 overexpression in the NSCs promoted their differentiation into neurons more than the astrocytes

Recently, due to the stability, low immunogenicity, and high safety profile following infection, lentivirus-transfected gene therapy has become a reliable and powerful technique in mediating the targeted gene expression both in vitro and in vivo studies ([20, 27, 36, 53]). DPYSL2 is known to be co-coded with a member of the collapsin response mediator protein family, in which proteins form homo- and hetero-tetramers enhance neuron guidance, growth, and polarity [19]. DPYSL2 was also identified as a mediator of axonal outgrowth in the developing brain via enhancement of microtubule assembly, vesicle trafficking, and synaptic physiology [28]. Overexpression of DPYSL2 protein in the hippocampal neurons promotes the formation of Ca^2+^ channel, while lentivirus-mediated DPYSL2 knockdown contrarily reverses this effect [7]. Similarly, to further demonstrate the role of DPYSL2 in the cultured NSCs and explore whether it could successfully promote the NSC differentiation into neurons, lentivirus transfection to overexpress DPYSL2 was successfully carried out in rats’ P2 NSCs. We found that DPYSL2 overexpression promotes NSCs’ differentiation into neurons more than the astrocytes.

### DPYSL2 depletion in the NSCs hindered their differentiation into neurons

In the central nervous system, DPYSL2 expression is concentrated at synaptic sites and axons. It was thereby assumed to impact synaptic physiology [3]. It has also been linked to numerous neurological disorders such as depression in the schizophrenia patients’ brain [26]. In experiments investigating the role of DPYSL2, destabilized anaphase astral microtubules together with altered spindle positions were exhibited in DPYSL2-depleted cells [22]. The absence of DPYSL2 expression may trigger neurodevelopmental disorders including unregulated axon growth and branching, indicating its crucial role in the pathogenesis of schizophrenia [26]. Correspondingly, with these results, we have observed the declined differentiation capacity of NSCs into neurons after knocking out DPYSL2 expression, which suggests that DPYSL2 plays an indispensable role in the nervous system function and disorders. To our knowledge, this study is the first to identify the association between DPYSL2 expression and NSC differentiation.

## Conclusion

These findings suggest that PNS promotes NSC differentiation into neurons and is associated with DPYSL2 overexpression. Taken together with functional evidence, the promotion of NSC differentiation into neurons closely correlates with the elevation of DPYSL2 expression, indicating tremendous progress in DPYSL2-based gene and NSC therapy for clinical nervous diseases.

## Data Availability

The datasets analyzed during the current study are available from the corresponding author on reasonable request.

## Abbreviations

PNS: *Panax notoginseng* saponins
NSCs: Neural stem cells
DPYSL2: Dihydropyrimidinase-like 2
+/+: Wild-type rats
−/−: DPYSL2-knockout rats
Con: Control group
Ser: Serum group
P + S: *Panax notoginseng* saponins and serum group

## References

[1] Ahmed AI, Gajavelli S, Spurlock MS, Chieng LO, Bullock MR. Stem cells for therapy in TBI. J R Army Med Corps. 2015;162(2):98–102. 10.1136/jramc-2015-000475 26338987

[2] Akama K, Horikoshi T, Nakayama T, Otsu M, Imaizumi N, Nakamura M, Toda T, Inuma M, Hirano H, Kondo Y, Suzuki Y, Inoue N. (2011). Proteomic identification of differentially expressed genes in neural stem cells and neurons differentiated from embryonic stem cells of cynomolgus monkey (Macacafascicularis) in vitro. BiochimBiophysActa 1814:265–276. 10.1016/j.bbapap.2010.10.009 21047566

[3] American Psychiatric Association (1994). Diagnostic and Statistical Manual of Mental Disorders.

[4] Arimura N, Hattori A, Kimura T, Nakamuta S, Funahashi Y, Hirotsune S, Furuta K, Urano T, Toyoshima YY, Kaibuchi K (2009). CRMP-2 directly binds to cytoplasmic dynein and interferes with its activity. J Neurochem.

[5] Bond AM, Peng CY, Meyers EA, McGuire T, Ewaleifoh O, Kessler JA (2014). BMP signaling regulates the tempo of adult hippocampal progenitor maturation at multiple stages of the lineage. Stem Cells.

[6] Brekk OR, Makridakis M, Mavroeidi P, Vlahou A, Xilouri M, Stefanis L (2019). Impairment of chaperone-mediated autophagy affects neuronal homeostasis through altered expression of DJ-1 and CRMP-2 proteins. Mol Cell Neurosci.

[7] Brittain JM, Piekarz AD, Wang Y (2009). An atypical role for collapsin response mediator protein 2 (CRMP-2) in neurotransmitter release via interaction with presynaptic voltage-gated calcium channels. J BiolChem.

[8] Butterfield DA, Perluigi M, Sultana R (2006). Oxidative stress in Alzheimer’s disease brain: new insights from redox proteomics. Eur J Pharmacol.

[9] Cai BX, Li XY, Chen JH, Tang YB, Wang GL, Zhou JG, Qui QY, Guan YY (2009). Ginsenoside-Rd, a new voltage-independent Ca2+ entry blocker, reverses basilar hypertrophic remodeling in stroke-prone renovascular hypertensive rats. Eur J Pharmacol.

[10] Chen X, Zhou M, Li Q, Yang J, Zhang Y, Zhang D, Kong S, Zhou D, He L. Sanchi for acute ischaemicstroke, Cochrane Database Syst Rev. 2008;(4):CD006305. 10.1002/14651858.CD006305.pub2 18843711

[11] Cui J, Jiang L, Xiang H (2012). Ginsenoside Rb3 exerts antidepressant-like effects in several animal models. J Psychopharmacol.

[12] Cui Y, Yin Y, Xiao Z, Zhao Y, Chen B, Yang B, Dai J (2019). LncRNA Neat1 mediates miR-124-induced activation of Wnt/beta-catenin signaling in spinal cord neural progenitor cells. Stem Cell Res Ther.

[13] Daynac M, Morizur L, Chicheportiche A, Mouthon MA, Boussin FD (2016). Age-related neurogenesis decline in the subventricular zone is associated with specific cell cycle regulation changes in activated neural stem cells. Sci Rep.

[14] Dooley D, Vidal P, Hendrix S (2014). Immunopharmacological intervention for successful neural stem cell therapy: new perspectives in CNS neurogenesis and repair. PharmacolTher.

[15] Esmaeilpour T, Fereydouni E, Dehghani F, Bokkon I, Panjehshahin MR, Csaszar-Nagy N, . . .Salari V. (2020). An experimental investigation of UltraweakPhoton emission from adult murine neural stem cells. Sci Rep, 10(1):463. 10.1038/s41598-019-57352-4 PMC6965084 31949217

[16] Fei T, Xia K, Li Z, Zhou B, Zhu S, Chen H, Zhang J, Chen Z, Xiao H, Han JD, Chen YG (2010). Genome-wide mapping of SMAD target genes reveals the role of BMP signaling in embryonic stem cell fate determination. Genome Res.

[17] Fu HZ, Zhong RJ, Zhang DM, Wang D (2013). A new protopanaxadiol-type ginsenoside from the roots of Panax notoginseng. J Asian Nat Prod Res.

[18] Gellert M, Venz S, Mitlohner J, Cott C, Hanschmann EM, Lillig CH (2013). Identification of a dithiol-disulfide switch in collapsin response mediator protein 2 (CRMP2) that is toggled in a model of neuronal differentiation. J BiolChem.

[19] Goshima Y, Nakamura F, Strittmatter P, Strittmatter SM (1995). Collapsin-induced growth cone collapse mediated by an intracellular protein related to UNC-33. Nature.

[20] Honda M, Minami I, Tooi N, Morone N, Nishioka H, Uemura K, Kinoshita A, Heuser JE, Nakatsuji N, Aiba K (2016). The modeling of Alzheimer’s disease by the overexpression of mutant Presenilin 1 in human embryonic stem cells. BiochemBiophys Res Commun.

[21] Huang JW, Du YQ, Li CJ, Yang JZ, Ma J, Zang YD, et al. Neuroprotectivetriterpenesaponins from the leaves of Panax notoginseng. Nat Prod Res. 2019;22:1–7.

[22] Hwayoung L, JaesoonJoo S-SN, Kim JW, Kim H-K, Kwon J-T, Lee H-Y, Kim YO, Kim H-J (2015). Changes in Dpysl2 expression are associated with prenatally stressed rat offspring and susceptibility to schizophrenia in humans. Int J Mol Med.

[23] Imperlini E, Orru S, Corbo C, Daniele A, Salvatore F (2014). Altered brain protein expression profiles are associated with molecular neurological dysfunction in the PKU mouse model. J Neurochem.

[24] Indraswari F, Wong PT, Yap E, Ng YK, Dheen ST (2009). Upregulation of Dpysl2 and Spna2 gene expression in the rat brain after ischemic stroke. NeurochemInt.

[25] Khanna R, Wilson SM, Brittain JM, Weimer J, Sultana R, Butterfield A, Hensley K (2012). Opening Pandora’s jar: a primer on the putative roles of CRMP2 in a panoply of neurodegenerative, sensory and motor neuron, and central disorders. Future Neurol.

[26] Johnston-Wilson NL, Sims CD, Hofmann JP, et al: Disease-specific alterations in frontal cortex brain proteins in schizophrenia, bipolar disorder, and major depressive disorder. The Stanley Neuropathology Consortium. Mol Psychiatry 5: 142–149, 2000. 10.1038/sj.mp.4000696 10822341

[27] Kim DH, Rossi JJ (2007). Strategies for silencing human disease using RNA interference. Nat Rev Genet.

[28] Lee PR, Brady DL, Shapiro RA (2007). Prenatal stress generates deficits in rat social behavior: reversal by oxytocin. Brain Res.

[29] Li Q, Liang X, Yang Y, Zeng X, Zhong X, Huang C (2020). Panax notoginseng saponins ameliorate cisplatin-induced mitochondrial injury via the HIF-1alpha/mitochondria/ROS pathway. FEBS Open Bio.

[30] Liu JW, Tian SJ, de Barry J, Luu B (2007). Panaxadiol glycosides that induce neuronal differentiation in neurosphere stem cells. J Nat Prod.

[31] Lu P, Jones LL, Snyder EY, Tuszynski MH (2003). Neural stem cells constitutively secrete neurotrophic factors and promote extensive host axonal growth after spinal cord injury. ExpNeurol.

[32] Liu S, Chen Z (2019). Employing endogenous NSCs to promote recovery of spinal cord injury. Stem Cells Int.

[33] Luo FC, Wang SD, Qi L, Song JY, Lv T, Bai J (2011). Protective effect of panaxatriol saponins extracted from Panax notoginseng against MPTP-induced neurotoxicity in vivo. J Ethnopharmacol.

[34] Nada SE, Shah ZA (2012). Preconditioning with Ginkgo biloba (EGb 761(R)) provides neuroprotection through HO1 and CRMP2. Neurobiol Dis.

[35] Nieber F, Hedderich M, Jahn O, Pieler T, Henningfeld KA (2013). NumbL is essential for Xenopus primary neurogenesis. BMC DevBiol.

[36] Qi YH, Yao WL, Zhang CH, Guo YQ (2014). Effect of lentivirus-mediated RNA interference of APC-Cdh1 expression on spinal cord injury in rats. Genet Mol Res.

[37] Si Y, Zhu J, Huang X, Zhu P, Xie C. Effects of Panax notoginseng saponinson proliferation and differentiation of rat embryonic cortical neural stem cells. J Chin Med Assoc. 2016;79(5):256–63. 10.1016/j.jcma.2015.10.011 26915440

[38] Si YC, Zhang JP, Xie CE, Zhang LJ, Jiang XN (2011). Effects of Panax notoginseng saponins on proliferation and differentiation of rat hippocampal neural stem cells. Am J Chin Med.

[39] Sirko S, Irmler M, Gascon S, Bek S, Schneider S, Dimou L, Obermann J, De Souza Paiva D, Poirier F, Beckers J, Hauck SM, Barde YA, Gotz M (2015). Astrocyte reactivity after brain injury-: the role of galectins 1 and 3. Glia.

[40] Song H, Wang P, Liu J, Wang C (2017). Panax notoginseng preparations for unstable angina pectoris: a systematic review and meta-analysis. Phytother Res.

[41] Song P, Xia X, Han T, Fang H, Wang Y, Dong F, Shen C. BMSCs promote the differentiation of NSCs into oligodendrocytes via mediating Id2 and Olig expression through BMP/Smad signaling pathway. Biosci Rep. 2018;38(5):BSR20180303. 10.1042/BSR20180303 PMC6147919 30143582

[42] Sun X, Gao RL, Lin XJ, Xu WH, Chen XH (2013). Panax notoginseng saponins induced up-regulation, phosphorylation and binding activity of MEK, ERK, AKT, PI-3K protein kinases and GATA transcription factors in hematopoietic cells. Chin J Integr Med.

[43] Sun Y, Fei T, Yang T, Zhang F, Chen YG, Li H, Xu Z (2010). The suppression of CRMP2 expression by bone morphogenetic protein (BMP)-SMAD gradient signaling controls multiple stages of neuronal development. J BiolChem.

[44] Tarn WY, Kuo HC, Yu HI, Liu SW, Tseng CT, Dhananjaya D, Hung KY, Tu CC, Chang SH, Huang GJ, Chiu IM. RBM4 promotes neuronal differentiation and neurite outgrowth via modulating Numb isoform expression. MolBiol Cell. 2016;27(10):1676–83. 10.1091/mbc.E15-11-0798 PMC4865323 27009199

[45] Than-Trong E, Ortica-Gatti S, Mella S, Nepal C, Alunni A, Bally-Cuif L. Neural stem cell quiescence and stemness are molecularly distinct outputs of the Notch3 signalling cascade in the vertebrate adult brain. Development. 2018;145(10):dev161034. 10.1242/dev.161034 PMC6001379 29695612

[46] Wakabayashi T, Hidaka R, Fujimaki S, Asashima M, Kuwabara T (2014). MicroRNAs and epigenetics in adult neurogenesis. Adv Genet.

[47] Wang Z, Wang Y, Zhao J, Gutkind JS, Srivatsan A, Zhang G, Liao HS, Fu X, Jin A, Tong X, Niu G, Chen X (2015). Polymeric nanovehicle regulated spatiotemporal real-time imaging of the differentiation dynamics of transplanted neural stem cells after traumatic brain injury. ACS Nano.

[48] Wu T, Jia Z, Dong S, Han B, Zhang R, Liang Y (2019). Panax notoginseng saponins ameliorate leukocyte adherence and cerebrovascular endothelial barrier breakdown upon ischemia-reperfusion in mice. J Vasc Res.

[49] Xie W, Meng X, Zhai Y, Zhou P, Ye T, Wang Z, et al. Panax notoginseng saponins: a review of its mechanisms of antidepressant or anxiolytic effects and network analysis on phytochemistry and pharmacology. Molecules. 2018;23(4):940. 10.3390/molecules23040940 PMC6017639 29673237

[50] Yang Q, Wu J, Zhao J, Xu T, Zhao Z, Song X, Han P (2018). Circular RNA expression profiles during the differentiation of mouse neural stem cells. BMC SystBiol.

[51] Yang YC, Liu BS, Shen CC, Lin CH, Chiao MT, Cheng HC (2011). Transplantation of adipose tissue-derived stem cells for treatment of focal cerebral ischemia. CurrNeurovasc Res.

[52] Ying Y, Zhang YL, Ma CJ, Li MQ, Tang CY, Yang YF, Shu XS (2019). Neuroprotective effects of ginsenoside Rg1 against hyperphosphorylated tau-induced diabetic retinal neurodegeneration via activation of IRS-1/Akt/GSK3beta signaling. J Agric Food Chem.

[53] Younis A, Siddique MI, Kim CK, Lim KB (2014). RNA Interference (RNAi) Induced Gene Silencing: A Promising Approach of Hi-Tech Plant Breeding. Int J BiolSci.

[54] Zhang Z, Xu G, Cai B, Zhang H, Zhu W, Liu X. Genetic variants in microRNAs predict recurrence of ischemic stroke. MolNeurobiol. 2016;54(4):2776–2780. 10.1007/s12035-016-9865-7 27011381

[55] Zhou L, Huang PP, Chen LL, Wang P (2019). Panax notoginseng saponins ameliorate abeta-mediated neurotoxicity in C elegans through antioxidant activities. Evid Based Complement Alternat Med.

